# Exercise-induced pulse pressure augmentation is positively associated with exercise capacity in patients after transcatheter aortic valve implantation

**DOI:** 10.3389/fphys.2026.1896349

**Published:** 2026-08-03

**Authors:** Kotaro Takizawa, Daichi Kobayashi, Shinya Tajima, Junko Sakamoto, Kentaro Hori, Atsuko Nakayama

**Affiliations:** 1 Department of Rehabilitation, Sakakibara Heart Institute, Tokyo, Japan; 2 Department of Cardiology, Sakakibara Heart Institute, Tokyo, Japan

**Keywords:** blood pressure response, cardiopulmonary exercise testing, elderly, exercise capacity, pulse pressure, transcatheter aortic valve implantation

## Abstract

Although transcatheter aortic valve implantation (TAVI) improves outcomes in elderly patients with aortic stenosis, many continue to have reduced exercise capacity. While higher resting pulse pressure (PP) has been linked to worse prognosis, the impact of exercise-induced PP changes remains unclear. This study examined the association between PP change (ΔPP) during exercise and exercise capacity after TAVI. Methods: This single-center retrospective study included patients who underwent transfemoral TAVI at the Sakakibara Heart Institute between 2014 and 2024 and subsequently participated in outpatient cardiac rehabilitation. ΔPP was defined as the difference in PP between rest and peak oxygen uptake (peak VO_2_) during cardiopulmonary exercise testing. The association between ΔPP and exercise capacity (peak VO_2_) was evaluated using multivariable linear regression analyses. Results: A total of 71 patients were analyzed (median age 82 years (range 70–92), 46% women). The High ΔPP group (ΔPP > 47 mmHg, n = 35) showed significantly higher peak VO_2_ than the Low ΔPP group (19.8 vs. 15.6 mL/kg/min, *p* < 0.01). In multivariable linear regression models adjusted for age and sex, widened ΔPP remained associated with higher exercise capacity (coefficient 0.55 per 10 mmHg; 95% confidence interval 0.06–1.04; *p* = 0.029). Conclusions: In this single-center retrospective cohort, greater exercise-induced ΔPP was associated with higher peak VO_2_ after TAVI. The physiological determinants and clinical utility of this association require prospective validation.

## Introduction

Transcatheter aortic valve implantation (TAVI) has become a standard treatment option for patients with severe aortic stenosis, particularly those who are elderly or have comorbidities that make conventional open-heart surgery challenging, and it has been shown to improve survival and symptoms (UK TAVI Trial Investigators, 2022; Takemoto et al., 2025). However, many patients continue to exhibit reduced exercise capacity after TAVI, which has been reported to be associated with increased all-cause mortality and cardiovascular events (Abdul-Jawad Altisent et al., 2017; van Erck et al., 2022; Cunha et al., 2022).

Previous studies have identified background factors such as age, female sex, and comorbid chronic obstructive pulmonary disease (COPD) as being associated with impaired exercise capacity after TAVI (Abdul-Jawad Altisent et al., 2017; Mok et al., 2013a). From a clinical perspective, it has also been reported that higher discharge systolic blood pressure (SBP) and pulse pressure (PP)—with SBP remaining significant in multivariable analyses—were associated with lower exercise capacity as assessed by the 6-minute walk test (Reinthaler et al., 2014).

Although these findings were derived from single resting measurements, studies in patients with heart failure with preserved ejection fraction (HFpEF) have shown that exercise-induced PP augmentation (i.e., PP/SBP) is associated with an increased risk of cardiovascular events (Namasivayam et al., 2022). These data suggest that blood pressure responses during exercise may also have distinct implications for exercise capacity in patients after TAVI, compared with those observed at rest. Exercise blood pressure responses are routinely measurable and may provide complementary physiological information beyond resting measurements. Thus, focusing on hemodynamic parameters such as SBP and PP provides an important applied perspective. Nevertheless, no priorstudy has directly examined the relationship between pulse pressure changes and exercise capacity in patients after TAVI.

We therefore sought to clarify the relationship between exercise-induced ΔPP and exercise capacity, as assessed by peak oxygen uptake (peak VO_2_), in patients after TAVI.

## Methods

### Subjects

**Figure 1:**
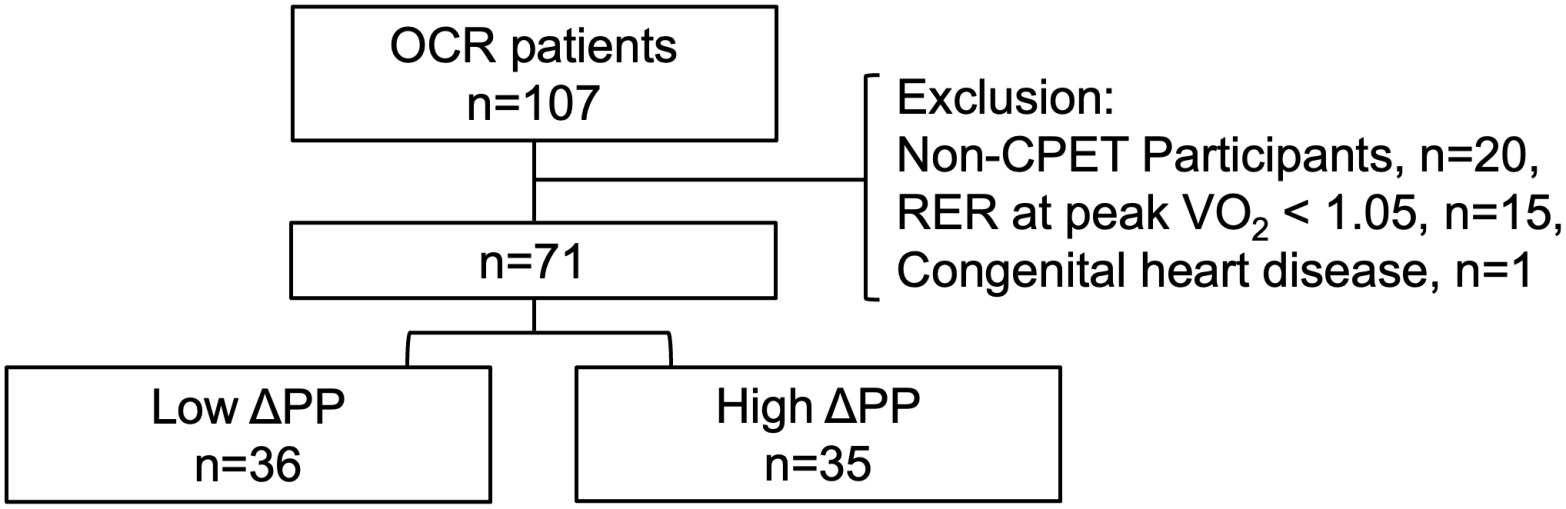
Flow diagram of patient inclusion and exclusion. CPET, cardiopulmonary exercise testing; ΔPP, change in pulse pressure (Peak − Rest); OCR, outpatient cardiac rehabilitation; RER, respiratory exchange ratio.

This single-center retrospective study was conducted at the Sakakibara Heart Institute, one of Japan’s leading cardiovascular centers. Patients who underwent transfemoral TAVI at the institution and subsequently entered outpatient cardiac rehabilitation between January 2014 and December 2024 were screened for eligibility (**Figure 1**). Exclusion criteria were: (1) no performance of cardiopulmonary exercise testing (CPET), (2) respiratory exchange ratio (RER) < 1.05 at peak VO_2_, and (3) congenital heart disease. The study was approved by the Institutional Review Board of the Sakakibara Heart Institute (IRB Approval No. 25-034). Given the retrospective design and the use of de-identified data, the requirement for individual informed consent was waived in accordance with institutional guidelines.

### CPET protocol and measurements

Cardiopulmonary exercise testing (CPET) was performed using a cycle ergometer (StrengthErgo 8; Mitsubishi Electric Engineering Co., Ltd., Tokyo, Japan). After a 4-minute rest on the ergometer, exercise was initiated with a 4-minute warm-up at either 0 or 20 W. Thereafter, the work rate was increased by 10 or 20 W per minute until the patient reached symptom limitation or met predefined termination criteria, including exhaustion, angina, hypotension or hypertension, ST-segment changes, or arrhythmia. For elderly patients or those without prior exercise habits before the procedure, a lower-intensity protocol was adopted: after a 4-minute warm-up at 0 W, the work rate was increased by 10 W per minute to avoid reaching the anaerobic threshold during the warm-up.

Gas exchange variables, including oxygen uptake (VO_2_), carbon dioxide output (VCO_2_), ventilation (VE), and peak VO_2_ (mL/kg/min), were continuously measured using a breath-by-breath system (ML-9000; Fukuda Denshi Co., Ltd., Tokyo, Japan). In addition, standard exercise variables, such as heart rate (HR), blood pressure, and electrocardiogram (ECG), were recorded at rest and throughout exercise using non-invasive methods.

### Variable definitions

Pulse pressure (PP) was defined as systolic blood pressure (SBP) minus diastolic blood pressure (DBP). The change in systolic blood pressure (ΔSBP) and the change in diastolic blood pressure (ΔDBP) were defined as the differences between SBP or DBP at peak VO_2_ and those at rest, respectively. The change in pulse pressure (ΔPP) was calculated as the difference between PP at peak VO_2_ and PP at rest. The change in heart rate (ΔHR) was defined as the difference between heart rate at peak VO_2_ and that at rest. Exercise capacity was defined by peak VO_2_ (mL/kg/min), as measured during CPET at the beginning of outpatient cardiac rehabilitation (OCR). For descriptive purposes, patients were stratified into Low and High ΔPP groups based on the median ΔPP value to summarize baseline characteristics (**Table 1**), given that no established clinical cutoff values or standard reference ranges for exercise-induced ΔPP currently exist. However, to preserve statistical power and avoid arbitrary cut-offs, the primary association between ΔPP and exercise capacity was evaluated using continuous variables in univariable and multivariable linear regression models.

**Table 1:** Patient characteristics and clinical parameters after TAVI.

Variable	All (n=71)	Low ΔPP (n=36)	High ΔPP (n=35)	P-value
Characteristics				
Age, years, median (range)	82 (70-92)	83 (77-92)	81 (70-90)	0.032
Female, n (%)	33 (46)	23 (64)	10 (29)	0.004
Body Mass Index, kg/m2	22.8 ± 2.7	22.9 ± 2.9	22.7 ± 2.5	0.704
Days after TAVI	46 ± 22	47 ± 25	45 ± 19	0.685
EuroSCORE II, points	3.47 ± 2.10	4.09 ± 2.07	2.85 ± 2.00	0.016
Hypertension, n (%)	55 (77)	30 (83)	25 (71)	0.267
Dyslipidemia, n (%)	44 (62)	21 (58)	23 (66)	0.627
Diabetes mellitus, n (%)	21 (30)	9 (25)	12 (34)	0.443
Chronic kidney disease, n (%)	16 (23)	6 (17)	10 (29)	0.260
COPD, n (%)	1 (1)	1 (3)	0 (0)	1.000
NYHA class, n (%)				0.563
I/II	57 (80)/14 (20)	30 (83)/6 (17)	27 (77)/8 (23)	
LVEF, %	62 ± 6	62 ± 6	62 ± 7	0.792
Medical history, n (%)				
CABG/PCI	15 (21)	8 (22)	7 (20)	1.000
Pacemaker/ICD	4 (6)	3 (8)	1 (3)	0.614
Stroke	4 (6)	2 (6)	2 (6)	1.000
HF hospitalization	5 (7)	1 (3)	4 (11)	0.199
Laboratory data				
Hemoglobin, g/dL	12.0 ± 1.3	11.8 ± 1.0	12.2 ± 1.5	0.177
Albumin, g/dL	4.1 ± 0.3	4.1 ± 0.3	4.0 ± 0.3	0.746
eGFR, mL/min/1.73m2	58 ± 15	56 ± 16	59 ± 14	0.357
NT-proBNP, pg/mL	436 ± 459	512 ± 516	361 ± 398	0.185
CRP, mg/dL	0.49 ± 1.00	0.56 ± 1.26	0.42 ± 0.69	0.562
Medications, n (%)				
ACE-I/ARB	39 (55)	21 (58)	18 (51)	0.637
β-Blocker	15 (21)	9 (25)	6 (17)	0.563
Calcium channel blocker	36 (51)	22 (61)	14 (40)	0.098
Diuretics	10 (14)	7 (19)	3 (9)	0.307
Statin	46 (65)	22 (61)	24 (69)	0.621
Nutrition, Physical function, ADL				
MNA-SF, points	10 ± 1	10 ± 1	11 ± 1	0.185
SMI, kg/m2	6.6 ± 1.0	6.3 ± 1.0	6.8 ± 0.9	0.044
Grip, kg	23 ± 7	21 ± 6	25 ± 8	0.022
IKES, %BW	39 ± 12	35 ± 8	45 ± 13	<0.001
SPPB, points	12 ± 1	11 ± 1	12 ± 1	0.513
Barthel Index, points	100 ± 0	100 ± 0	100 ± 0	–

Values are presented as mean ± SD, median (range), or n (%).

ACE-I, angiotensin-converting enzyme inhibitor; ADL, activities of daily living; ARB, angiotensin II receptor blocker; CABG, coronary artery bypass grafting; CRP, C-reactive protein; ΔPP, change in pulse pressure (Peak − Rest); eGFR, estimated glomerular filtration rate; EuroSCORE II, European System for Cardiac Operative Risk Evaluation II; Grip, handgrip strength; HF, heart failure; ICD, implantable cardioverter defibrillator; IKES, isometric knee extension strength; LVEF, left ventricular ejection fraction; MNA-SF, Mini Nutritional Assessment–Short Form; NT-proBNP, N-terminal pro-B-type natriuretic peptide; NYHA, New York Heart Association functional class; PCI, percutaneous coronary intervention; SMI, skeletal muscle index; SPPB, Short Physical Performance Battery.

### Statistical analysis

Continuous variables were expressed as mean ± SD or median (range), and categorical variables as n (%). Group comparisons were performed using the Mann-Whitney U test, Student’s *t*-test, Fisher’s exact test, or *χ²* test, as appropriate. To examine the relationships between ΔPP and blood pressure components (ΔSBP and ΔDBP), linear regression models were applied, and regression lines were depicted in scatter plots. To further assess the association between ΔPP and peak VO_2_, multivariable linear regression analyses were conducted. First, we assessed the linearity of the association using restricted cubic spline (RCS) analysis with 4 knots to visually explore the continuous dose-response relationship between ΔPP and peak VO_2_. Although likelihood ratio tests comparing the RCS model with a linear model showed no statistically significant non-linearity (Model 1:*p* = 0.491; Model 2: *p* = 0.756; Model 3: *p* = 0.431), likely reflecting limited statistical power given the sample size, the RCS curve was retained to provide a descriptive, hypothesis-generating visualization of the continuous relationship. Given the absence of significant non-linearity, multivariable linear regression was used to quantify the association. Explanatory variables included ΔPP as the main predictor, adjusted as follows: Model 1 for age and sex to account for basic demographic characteristics; Model 2 for age and isometric knee extension strength (IKES) to assess whether the association was independent of skeletal muscle function; and Model 3 for age and ΔHR to examine whether the association between ΔPP and peak VO_2_ remained after accounting for chronotropic response. Statistical analyses were performed using SPSS software (version 21, IBM Corp., Armonk, NY, USA), except for restricted cubic spline regression analyses, which were performed using R software version 4.5.2 (The R Foundation for Statistical Computing, Vienna, Austria) with the ‘rms’ package.

## Results

### Patient characteristics and clinical parameters after TAVI

Baseline patient characteristics, laboratory data, medication use, and nutritional and physical function parameters are summarized in **Table 1**. The median age of the overall cohort was 82 years, and 46% were women. Compared with the High ΔPP group, patients in the Low ΔPP group were significantly older (83 vs. 81 years) and more frequently women (64% vs. 29%). No significant differences were observed between groups with respect to the number of days since TAVI, comorbidities, or left ventricular ejection fraction. Regarding laboratory findings, there were no significant between-group differences in renal function (eGFR), markers of heart failure severity (NT-proBNP), or inflammatory status (CRP). With respect to medications, no statistically significant differences were observed in the overall distribution, including calcium channel blockers. For nutritional and physical function parameters, the High ΔPP group exhibited significantly higher skeletal muscle index, grip strength, and knee extension strength compared with the Low ΔPP group.

### Resting and exercise hemodynamics

**Figure 2:**
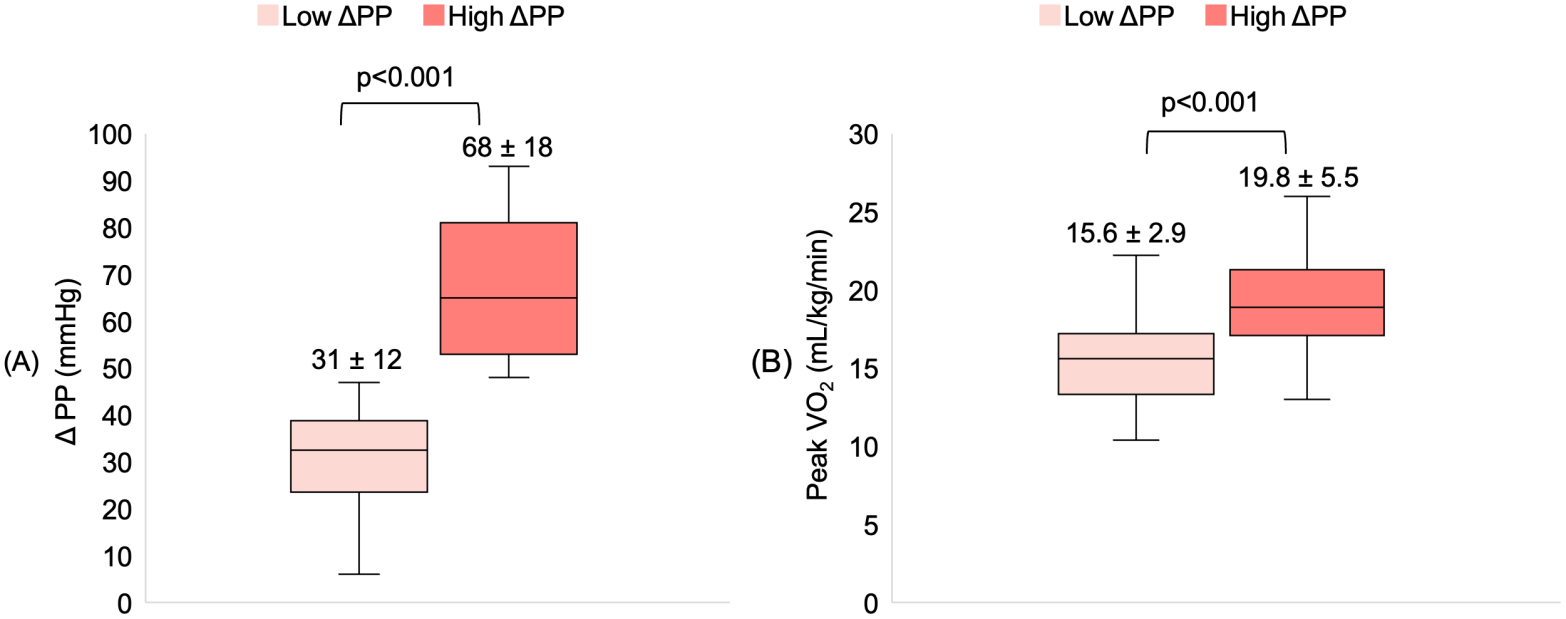
ΔPP **(A)** and peak VO_2_ **(B)** between low ΔPP and high ΔPP groups. The vertical axes represent **(A)** ΔPP (mmHg) and **(B)** peak VO_2_ (mL/kg/min). The horizontal axes indicate patient groups, defined by the median ΔPP during exercise. Box plots show the median with interquartile ranges, and whiskers represent the range. The lighter boxes represent the Low ΔPP group, and the darker boxes represent the High ΔPP group. ΔPP, change in pulse pressure (Peak − Rest).

**Figure 3:**
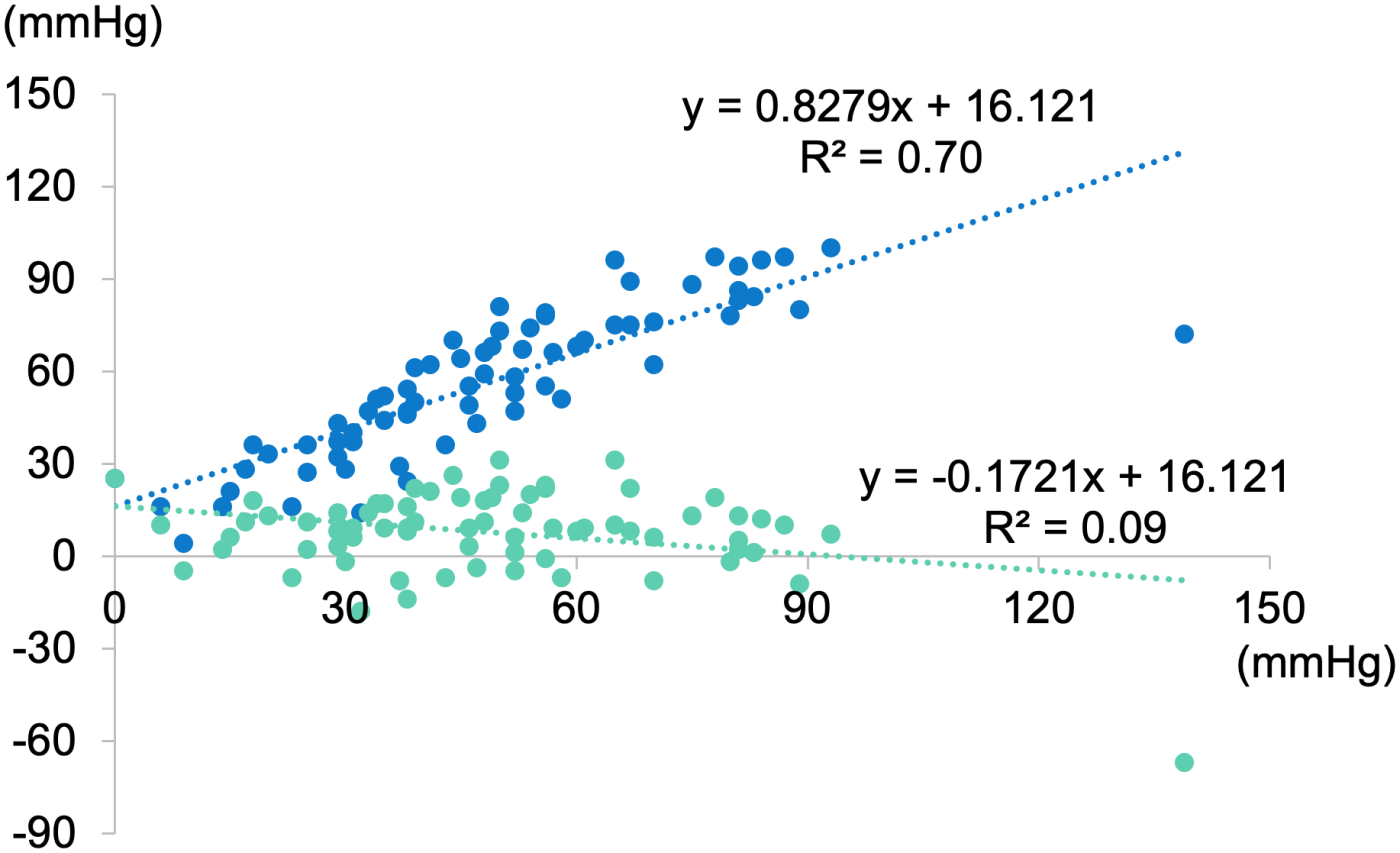
Correlation between ΔPP and ΔSBP/ΔDBP. The vertical axis represents ΔPP (mmHg), and the horizontal axis represents ΔSBP and ΔDBP (mmHg). Blue dots indicate ΔSBP values, and green dots indicate ΔDBP values. ΔDBP, change in diastolic blood pressure (Peak − Rest); ΔPP, change in pulse pressure (Peak − Rest); ΔSBP, change in systolic blood pressure (Peak − Rest).

Results of CPET are summarized in **Table 2**. At rest, systolic blood pressure was significantly higher in the Low ΔPP group. At peak exercise, however, the High ΔPP group exhibited significantly higher systolic blood pressure and PP. Respiratory efficiency, assessed by the VE/VCO_2_ slope, did not differ significantly between the groups. As shown in **Figure 2**, peak VO_2_, the primary outcome reflecting exercise capacity, was significantly higher in the High ΔPP group compared with the Low ΔPP group (19.8 vs. 15.6 mL/kg/min, *p* < 0.001). Similarly, the anaerobic threshold (AT), a submaximal index of exercise capacity, was also significantly higher in the High ΔPP group (12.6 vs. 11.0 mL/kg/min, *p* = 0.009). O_2_ pulse at AT was also significantly higher in the High ΔPP group (8.0 ± 2.0 vs. 6.6 ± 1.3 mL/beat, p = 0.001). In addition, ΔPP itself was significantly higher in the High ΔPP group than in the Low ΔPP group (68 ± 18 vs. 31 ± 12 mmHg, *p* < 0.001). O_2_ pulse at peak exercise was significantly higher in the High ΔPP group (9.4 ± 2.6 vs. 7.6 ± 1.5 mL/beat, p < 0.001). Peak RER was significantly higher in the High ΔPP group (1.24 ± 0.11 vs. 1.16 ± 0.08, p = 0.002), confirming that both groups achieved adequate maximal effort. **Figure 3** depicts the relationships between ΔPP and blood pressure components. ΔPP showed a strong positive correlation with ΔSBP (r = 0.84, *p* < 0.001) and a weak negative correlation with ΔDBP (r = −0.32, *p* = 0.007).

**Table 2:** Resting and exercise hemodynamics.

Variable	All (n=71)	Low ΔPP (n=36)	High ΔPP (n=35)	P-value
Rest				
HR, bpm	70 ± 11	71 ± 12	70 ± 11	0.824
SBP, mmHg	136 ± 20	142 ± 19	129 ± 19	0.005
DBP, mmHg	68 ± 11	70 ± 9	66 ± 12	0.096
PP, mmHg	68 ± 21	72 ± 21	63 ± 19	0.079
AT				
HR, bpm	91 ± 12	91 ± 12	92 ± 13	0.753
SBP, mmHg	165 ± 24	162 ± 22	167 ± 26	0.315
DBP, mmHg	73 ± 13	75 ± 11	71 ± 14	0.176
PP, mmHg	92 ± 26	87 ± 27	97 ± 25	0.119
VO_2_, mL/kg/min	11.8 ± 2.6	11.0 ± 1.9	12.6 ± 3.0	0.009
O_2_ pulse, mL/beat	7.3 ± 1.8	6.6 ± 1.3	8.0 ± 2.0	0.001
Peak				
HR, bpm	119 ± 17	115 ± 17	122 ± 18	0.079
SBP, mmHg	192 ± 25	180 ± 22	204 ± 22	<0.001
DBP, mmHg	76 ± 14	78 ± 13	74 ± 15	0.227
PP, mmHg	116 ± 26	103 ± 22	131 ± 23	<0.001
VO_2_, mL/kg/min	17.7 ± 4.8	15.6 ± 2.9	19.8 ± 5.5	<0.001
O_2_ pulse, mL/beat	8.5 ± 2.3	7.6 ± 1.5	9.4 ± 2.6	<0.001
ΔPP, mmHg	49 ± 24	31 ± 12	68 ± 18	<0.001
VE/VCO2 slope	30 ± 8	32 ± 9	29 ± 7	0.152
ΔVO_2_/ΔWR, mL/min/Watt	8.9 ± 2.2	7.9 ± 1.7	10.0 ± 2.2	<0.001
RER	1.20 ± 0.10	1.16 ± 0.08	1.24 ± 0.11	0.002

Values are presented as mean ± SD, median.

AT, anaerobic threshold; DBP, diastolic blood pressure; ΔPP, change in pulse pressure (Peak − Rest); ΔVO_2_/ΔWR, oxygen uptake–work rate relationship; HR, heart rate; PP, pulse pressure; RER, respiratory exchange ratio; SBP, systolic blood pressure; VE/VCO_2_ slope, minute ventilation–carbon dioxide production slope.

### Association between widened pulse pressure and higher exercise capacity

**Figure 4:**
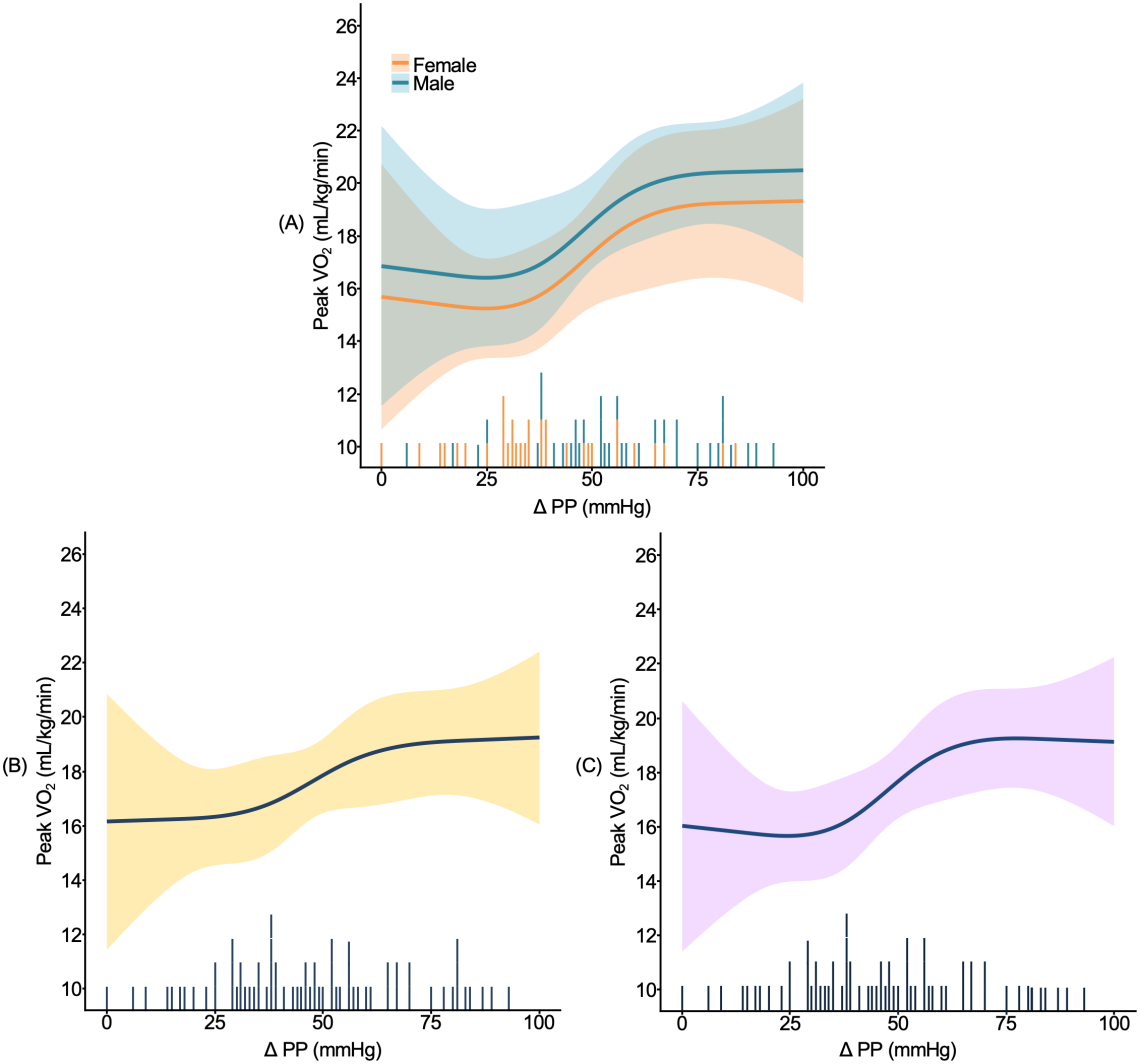
Association between ΔPP and peak VO_2_ using restricted cubic spline regression. **(A)** Model 1: Predicted Peak VO_2_ for females (orange) and males (blue), adjusted for age (mean: 82.4 years). **(B)** Model 2: Predicted Peak VO_2_ adjusted for age and IKES %BW (mean: 39.5%). **(C)** Model 3: Predicted Peak VO_2_ adjusted for age and Δ HR (mean: 48.2 bpm). Solid lines represent the estimates, and lighter shaded areas indicate 95% confidence intervals. Δ HR, change in heart rate (Peak − Rest); Δ PP, change in pulse pressure (Peak − Rest); HR, heart rate; IKES, isometric knee extension strength.

Restricted cubic spline analysis descriptively showed a steep increase in peak VO_2_ as ΔPP rose from approximately 40 to 60 mmHg, followed by a plateau at higher levels (**Figure 4**), with likelihood ratio tests confirming no significant non-linearity (all *p* > 0.05). Multivariable linear regression analyses (**Table 3**) showed that greater ΔPP during exercise was associated with higher peak VO_2_ after adjustment for age and sex (coefficient [β] 0.55; 95% confidence interval [CI] 0.06–1.04; *p* = 0.029). This association remained significant after additional adjustment for ΔHR in Model 3 (β 0.50; 95% CI 0.08–0.93; *p* = 0.021). In Model 2, although the association fell just short of statistical significance after adjusting for isometric knee extension strength (β 0.40; 95% CI -0.04–0.85; *p* = 0.076), the direction of the association remained consistent.

**Table 3:** Multivariable linear regression analyses for exercise capacity.

Variable	Coefficient (β)	95% CI	P-value
Model 1			
ΔPP, per 10 mmHg	0.55	0.06 - 1.04	0.029
Age, per 5 years	-0.94	-2.38 - 0.50	0.198
Male sex	1.50	-0.86 - 3.86	0.209
Model 2			
ΔPP, per 10 mmHg	0.40	-0.04 - 0.85	0.076
Age, per 5 years	-0.66	-2.00 - 0.69	0.332
IKES (%BW)	0.16	0.06 - 0.25	0.001
Model 3			
ΔPP, per 10 mmHg	0.50	0.08 - 0.93	0.021
Age, per 5 years	-0.74	-2.09 - 0.62	0.282
ΔHR, per 10 bpm	1.12	0.51 - 1.74	<0.001

Values are unstandardized regression coefficients (β) with 95% confidence intervals (CI), derived from linear regression models. Model 1: adjusted for age and sex. Model 2: adjusted for age and IKES. Model 3: adjusted for age and ΔHR.

%BW, percent of body weight; CI, confidence interval; ΔHR, change in heart rate (Peak − Rest); ΔPP, change in pulse pressure (Peak − Rest); IKES, isometric knee extension strength.

## Discussion

This study is one of the few to evaluate exercise capacity using CPET in elderly patients after TAVI, and identified a positive association between exercise-induced ΔPP and peak VO_2_. Exercise-induced ΔPP may be viewed as a candidate integrated exercise hemodynamic marker related to cardiovascular and peripheral factors; however, the present data do not establish its physiological determinants. The association remained after adjustment for age and sex and after adjustment for age and ΔHR, but was attenuated after adjustment for IKES. These findings should therefore be interpreted as hypothesis-generating and require prospective mechanistic validation. Importantly, this relationship remained significant after adjusting for age and sex (Model 1), as well as for changes in heart rate (Model 3), suggesting that ΔPP may carry hemodynamic information that is not fully explained by chronotropic response. It is noteworthy that in Model 2, the association between ΔPP and peak VO_2_ was attenuated and fell short of statistical significance (*p* = 0.076) after adjusting for isometric knee extension strength (IKES). We acknowledge that this attenuation is an important observation: skeletal muscle function and the muscle pump may contribute meaningfully to the ΔPP–peak VO_2_ relationship, possibly through enhanced venous return and integrated peripheral circulatory adaptation during exercise. This is consistent with the robust independent effect of IKES on exercise capacity observed in our cohort (*p* = 0.001) and in prior studies in elderly populations (Haykowsky et al., 2011; Lang et al., 1997; Kamiya et al., 2014). The possibility that ΔPP partly reflects skeletal muscle reserve and venous return rather than cardiac contractile reserve alone cannot therefore be excluded. Nevertheless, the regression coefficient for ΔPP remained positive and directionally consistent across all models, suggesting that ΔPP may still capture hemodynamic information beyond muscle strength alone, although the current sample size does not permit firm mechanistic separation.

The observed ΔPP augmentation during exercise likely reflects a combination of cardiovascular and peripheral factors. Because pulse pressure is determined by the interplay of stroke volume and arterial compliance (Homan et al., 2025), its exercise-induced rise could indicate augmented stroke volume, reduced arterial stiffness, or both. However, we acknowledge that arterial compliance was not measured in this study and that patients may differ in vascular properties independently of cardiac function. Furthermore, direct measures of ventricular contractility, cardiac output, or ventriculo-arterial coupling were not available. These unmeasured variables represent important limitations in the mechanistic interpretation of our findings. O_2_ pulse was higher in the High ΔPP group at both peak exercise (9.4 ± 2.6 vs. 7.6 ± 1.5 mL/beat, *p* < 0.001) and at the anaerobic threshold (8.0 ± 2.0 vs. 6.6 ± 1.3 mL/beat, *p* = 0.003). Because O_2_ pulse is influenced by both stroke volume and peripheral oxygen extraction, these findings are compatible with differences in integrated oxygen delivery but do not establish that ΔPP reflects cardiac or stroke volume reserve. In addition, peak RER was significantly higher in the High ΔPP group (1.24 ± 0.11 vs. 1.16 ± 0.08, *p* = 0.002), indicating that both groups achieved adequate maximal effort and that the difference in peak VO_2_ is unlikely to reflect differences in exercise effort alone. Although β-blocker use can suppress hemodynamic responses, no significant difference in prescription rates was observed between groups (**Table 1**), and the association between ΔPP and exercise capacity remained significant after adjusting for chronotropic response (ΔHR) in Model 3. These observations suggest that the ΔPP–peak VO_2_ association is not solely attributable to differences in heart rate augmentation.

Prior studies have linked elevated resting PP with reduced exercise capacity and poorer outcomes after TAVI. In contrast, our study focused on dynamic PP responses to exercise, which may represent a physiologically distinct marker. In our cohort, higher resting PP was observed in the Low ΔPP group, which exhibited lower exercise capacity, consistent with prior evidence, although the difference was not statistically significant. Thus, resting and exercise-induced PP may have distinct and complementary implications for functional status after TAVI. The absence of a significant between-group difference in the VE/VCO_2_ slope, together with more favorable physical function indices in the High ΔPP group, supports a cautious interpretation of the present findings. These observations suggest that the higher exercise capacity in the High ΔPP group may reflect not only cardiovascular reserve but also preserved peripheral function and lower frailty burden. This is consistent with reports that skeletal muscle oxygen utilization is a key determinant of exercise capacity in older adults (Haykowsky et al., 2011; Kondo et al., 2018) and that muscle strength modulates the blood pressure response during exercise (Van Daele et al., 2020). In addition, while patients with aortic stenosis often demonstrate a blunted SBP response during exercise (Badiani et al., 2025), our High ΔPP group exhibited a robust rise in SBP and significantly higher AT. Since peak VO_2_ can be influenced by patient motivation, the concurrent elevation of AT—a submaximal index less dependent on effort—suggests that the difference in peak VO_2_ was unlikely to be explained by exercise effort alone.

The prognostic role of blood pressure indices after TAVI has been inconsistently reported. In particular, Perlman et al. demonstrated that the post-procedural blood pressure response—rather than resting levels—was associated with improved cardiac function and fewer complications (Perlman et al., 2013), whereas Goldberg et al. found that acute SBP elevation predicted adverse cardiovascular outcomes at one year (Goldberg et al., 2024). Another study reported no significant association between periprocedural PP changes and prognosis (Parikh et al., 2022). Collectively, these discrepancies suggest that resting or peri-procedural elevations in SBP or PP do not consistently indicate a favorable prognosis. By contrast, our findings indicate that exercise-induced ΔPP is positively associated with exercise capacity, paralleling observations that postoperative SBP increases reflecting stroke volume augmentation were linked to better outcomes in some cohorts (Perlman et al., 2013; Nara et al., 2019).

Given the high prevalence of hypertension in this population, antihypertensive therapy is often necessary (Izumi et al., 2020; Vahanian et al., 2022; Otto et al., 2021). The present study does not establish whether modifying exercise-related ΔPP would improve functional capacity or prognosis after TAVI, and this question requires prospective investigation. Nevertheless, our findings raise the hypothesis that excessive pharmacological blood pressure lowering might potentially blunt the physiological rise in pulse pressure during exercise. Our restricted cubic spline analysis (**Figure 4**) provides hypothesis-generating data in this regard, suggesting a steep increase in peak VO_2_ with greater ΔPP augmentation up to a certain point, followed by a plateau. Whether preserving or optimizing exercise-induced ΔPP through individualized antihypertensive management could translate into improved functional outcomes remains to be determined in future prospective studies. We would emphasize that these implications are speculative based on the current retrospective data and should not be interpreted as practice-changing recommendations.

The novelty of this study lies in identifying exercise-induced ΔPP as a clinically accessible hemodynamic marker that is distinct from resting PP and may be obtainable during routine cardiac rehabilitation without specialized equipment. We propose that ΔPP is not a substitute for formal CPET or echocardiographic assessment, but may serve as a simple adjunctive marker to help identify patients with reduced functional reserve after TAVI, particularly in clinical settings where CPET is not readily available. In this context, a blunted ΔPP response during rehabilitation exercise may prompt closer functional assessment or more individualized exercise programming. Given the well-established link between exercise capacity and long-term outcomes (Myers et al., 2002; Keteyian et al., 2008; Mok et al., 2013b), incorporating exercise blood pressure responses into the routine clinical evaluation of post-TAVI patients represents a potentially practical and low-cost approach, though prospective validation is required before clinical implementation.

### Limitations

This study has several limitations. First, it was conducted at a single center with a relatively small sample size, which limits generalizability. Second, the study population was limited to patients capable of participating in outpatient rehabilitation and completing symptom-limited CPET with adequate effort (RER ≥ 1.05). Consequently, frailer patients or those with severe complications may have been implicitly excluded, and our findings might not fully apply to the broader, higher-risk post-TAVI population. Third, owing to its retrospective design, causal relationships cannot be established, and the present study does not support any specific change in clinical practice. Fourth, blood pressure was measured non-invasively using brachial cuffs. While this method enhances the applicability of our findings to real-world clinical settings, it reflects peripheral rather than central aortic pressure, which may differ due to arterial stiffness and wave reflection, particularly in this elderly cohort; future studies using central hemodynamic assessment may clarify whether central pulse pressure has a stronger relationship with exercise capacity. Fifth, echocardiographic parameters reflecting forward flow—such as stroke volume and stroke volume index—were not consistently available at a timepoint sufficiently aligned with CPET in our retrospective dataset. Therefore, the study cannot definitively determine whether exercise-induced ΔPP primarily reflects stroke volume reserve. Sixth, post-TAVI prosthetic or paravalvular aortic regurgitation may influence pulse pressure and could therefore affect the interpretation of the present findings. However, regurgitation grading data suitable for uniform analysis were not consistently available in our retrospective dataset, and this represents an important limitation. Seventh, arterial stiffness and compliance were not directly measured, and advanced hemodynamic parameters such as ventriculo-arterial coupling, arterial elastance, and ventricular contractility indices were not available. These unmeasured variables represent important limitations in the mechanistic interpretation of ΔPP. Eighth, detailed information regarding medication dose, timing, and exercise-specific pharmacologic interactions was not available in a standardized manner, which precludes a comprehensive assessment of the potential effects of antihypertensive therapy on exercise hemodynamics. Despite multivariable adjustment, residual and unmeasured confounding cannot be excluded, particularly from differences in the amount and intensity of rehabilitation before CPET, habitual physical activity, medication dose and timing, vascular stiffness, baseline frailty, and other clinical characteristics. In addition, the modest sample size limited the number of covariates that could be included in the regression models. Finally, this study evaluated exercise capacity as a functional outcome and did not assess hard clinical endpoints such as mortality, cardiovascular events, or heart failure hospitalization. Whether the exercise-induced ΔPP response is associated with long-term prognosis remains to be determined in prospective studies with adequate follow-up.

## Conclusions

In elderly patients after TAVI, pulse pressure augmentation in response to exercise was positively associated with exercise capacity as assessed by peak VO_2_. These findings suggest that dynamic changes in pulse pressure during exercise may provide clinically relevant information for patient evaluation after TAVI, beyond conventional resting measurements.

## Data Availability

The data underlying this article are available from the corresponding author upon reasonable request, subject to applicable ethical, institutional, and data protection requirements.
